# Comparative Study of Different Pretreatment and Combustion Methods on the Grindability of Rice-Husk-Based SiO_2_

**DOI:** 10.3390/nano13222951

**Published:** 2023-11-15

**Authors:** Yunhai Ma, Shengwang Yuan, Zichao Ma, Yihao Hou, Shichao Niu, Li Lekai, Guoqin Liu, Feipeng Cao

**Affiliations:** 1 Key Laboratory of Bionic Engineering (Ministry of Education, China), Jilin University, 5988 Renmin Street, Changchun 130022, China; ysw5968@163.com (S.Y.); houyihao825@163.com (Y.H.); niushichao@jlu.edu.cn (S.N.); lilk20@mails.jlu.edu.cn (L.L.); lgq1277993904@163.com (G.L.); 2 Institute of Structured and Architected Materials, Liaoning Academy of Materials, Shenyang 110167, China; caofp97@163.com; 3 Department of Mechanical Engineering, The Pennsylvania State University, State College, PA 16802-4400, USA; zvm5162@psu.edu

**Keywords:** rice husk, waste utilization, biomass combustion, Taguchi method, grindability, median diameter

## Abstract

The rice husk (RH) combustion pretreatment method plays a crucial role in the extraction of nanoscale SiO_2_ from RH as a silicon source. This study examined the effects of diverse pretreatment methods and combustion temperatures on the particle size distribution of nanoscale high-purity amorphous SiO_2_ extracted from rice husk ash (RHA) post RH combustion. The experiment was structured using the Taguchi method, employing an L9 (2^1^ × 3^3^) orthogonal mixing table. The median diameter (D50) served as the output response parameter, with the drying method (A), combustion temperature (B), torrefaction temperature (C), and pretreatment method (D) as the input parameters. The results showed the torrefaction temperature (C) as being the predominant factor affecting the D50, which decreased with an increasing torrefaction temperature (C). The optimal parameter combination was identified as A2B2C3D2. The verification test revealed that roasting could improve the abrasiveness of Rh-based silica and reduce the average particle size. Torrefaction at medium temperatures might narrow the size distribution range of RHA-SiO_2_. We discovered that the purity of silica increased with an increasing roasting temperature by evaluating the concentration of silica in the sample. The production of RHA with silica concentrations up to 92.3% was investigated. X-ray diffraction analysis affirmed that SiO_2_’s crystal structure remained unaltered across different treatment methods, consistently presenting as amorphous. These results provide a reference for extracting high-value products through RH combustion.

## 1. Introduction

China stands as the foremost producer and consumer of rice, churning out approximately 210 million tons annually. This production scale results in a consequent annual yield of rice husks (RHs) of 43.35 million tons, accounting for 28.9% of the global production [1,2]. Despite its abundance, RH’s challenging characteristics, such as its tough texture, high ash content, and low digestibility, mean that it is rarely used as soil fertilizer or livestock feed. Historically, vast quantities of RHs were either incinerated, left exposed, or relegated to waste filler roles [3]. Current SiO_2_ synthesis processes usually rely on high-cost silicate and silicon alkoxides as key raw materials [4,5,6], highlighting a pressing need to identify eco-friendly and cost-effective alternatives. Consequently, RH, a resource-rich and renewable material, contains a large amount of SiO_2_.

SiO_2_ serves as an important functional material, with versatile applications in environmental, industrial production, and biomedical fields [7]. Given the SiO_2_ richness of RH, there is a burgeoning interest in harnessing both RH and rice husk ash (RHA) as primary raw materials for SiO_2_ extraction [8]. Under relatively mild conditions, SiO_2_ can be separated from either RH or RHA, facilitating the production of a spectrum of SiO_2_ derivatives. The silica content in rice husk can vary from 5% to 30% depending on geographical location and the type of rice species used for the collected husk [9]. For instance, SiO_2_ and nano-silica can potentially replace commercial SiO_2_, thereby enhancing the mechanical properties of polymers when used as fillers [10].

RHA is the result of the direct combustion of RH in air at high temperatures, serving as a simple method for SiO_2_ production. However, challenges arise due to the difficulties in regulating the reaction temperature. This leads to a pronounced agglomeration, enlarging the particle size, and it is simple to inadvertently convert SiO_2_ from an amorphous to a crystalline structure [11]. As the combustion temperature increases, the K+ cation will reduce the crystallization temperature of the silica, causing it to merge with surrounding particles, thus forming larger particle aggregates [12]. Evidently, the pre-combustion removal of most metal cations from nano-silicon dioxide profoundly influences its purity and particle size [13,14].

Prior to combustion, RH undergoes processes like leaching and impurity elimination. The standard procedures encompass treatment methods with deionized water [15], organic acid [4], inorganic acid [16], and aqueous bio-oil [17]. In a comparative study of elemental removal, elements like K, Na, Mg, P, and Cl demonstrated higher removal efficiencies (up to 99.98%) through acid washing, especially when contrasted with metals such as Ca, Fe, Mn, and Cr [18]. A two-fold reduction in potassium content was witnessed [19]. While acid leaching for RH pretreatment method outperforms boiling-water leaching in purification efficacy, the latter proves to be more economical and eco-friendly [20,21]. Typically, acid leaching exhibits superior purification results compared to water leaching, with inorganic acids manifesting a more potent effect than their organic counterparts. Amorphous SiO_2_, following the acid-leaching pretreatment method, can achieve purities exceeding 95% [12,22]. This study employs a sulfuric acid solution.

Torrefaction can be regarded as a gradual combustion process under moderate conditions ranging between 200 and 300 °C. Post torrefaction, biomass exhibits a significant reduction in oxygen content, coupled with enhancements in calorific value, grindability, and hydrophobicity [20]. Several studies have shown that the torrefaction process can significantly improve the subsequent combustion, gasification, and combustion utility of biomass [23,24]. Consequently, pinpointing the suitable torrefaction temperature and its synergy with the pickling pretreatment method holds significant implications for the subsequent utilization of RH biomass combustion.

The exploration of biomass combustion or drying using microwave heating technologies is in its nascent stages. Unlike conventional heating methods, microwave heating originates heating intrinsically within the material, radiating outwards due to its distinct hotspot effect [25]. This technique can affect the crystalline grain’s growth process and modulate the material’s microstructure [26]. Furthermore, by swiftly escalating the material’s reaction temperature, the microwave treatment method can alter the crystalline grain growth pattern, preserving the material’s nanoscale structure [27]. However, while microwave heating curtails surface overheating, the potential for internal overheating persists, stemming from heat accumulation [28].

In this study, the effect of different pretreatment methods and combustion temperatures on the granulometric distribution of nanoscale amorphous silica extracted from RH was systematically investigated for the first time. The experimental variables encompassed the drying method, combustion temperature, torrefaction temperature, and pretreatment method. Employing a traditional full factorial design for these four variables would necessitate 162 experimental groupings, with each undergoing three repetitive tests. To ensure time efficiency and cost-effectiveness, this study adopted the Taguchi experimental design, known for its streamlined, effective, and systematic approach toward optimizing performance, quality, and cost [8]. The Taguchi method is a valuable engineering strategy for selecting optimal levels of processing parameters with minimal sensitivity to various causes of variability. The Taguchi method defines a process to evaluate which parameters, or controllable variables, have the most noise and influence on the objective function. The objective function is the system output intended to be optimized, maximized, or minimized [29,30]. The unique feature of the Taguchi method is that it can give adequate optimization results using relatively fewer experimental runs, making it more cost-efficient [31,32]. This experimental design used an L9 (2^2^ × 3^3^) orthogonal hybrid array, designating the median diameter (D50) as the output response metric, with the drying method, combustion temperature, torrefaction temperature, and pretreatment method as the input parameters. The findings of this study have significance for purifying nano-silicon dioxide, targeting high-end applications, and leveraging RH biomass as an eco-friendly SiO_2_ source.

## 2. Materials and Methods

### 2.1. Materials

RH samples were obtained from a rice-processing facility in Heilongjiang Province, China. Analytical-grade acetone, hydrofluoric acid, concentrated sulfuric acid, and sodium hydroxide were procured from Beijing Chemical Reagent Co., Ltd., Beijing, China. Before the experiments, the samples were dried using a blast dryer (Model: JF980B, Jilin Ji Da Electromechanical Equipment Co., Ltd., Changchun, China) at a temperature of 105 ± 5 °C for a duration of 24 h. Subsequently, these samples were packaged and stored in a drying dish for future use. Common pretreatment methods applied before RH combustion encompassed acid leaching, boiling, microwave exposure, and torrefaction. This study investigated the particle size distribution of RH-based silica prepared via different pretreatment and combustion methods. The approximate analysis of the sample was determined according to ASTM standards [33]. The proximal and ultimate analytical findings of the samples are presented in Table 1.

### 2.2. Preparation and Ball Milling of RHA-SiO_2_

The dried RH underwent a pretreatment method, as delineated in Table 2. The processes of torrefaction and combustion were executed sequentially in this experiment. Post torrefaction in the muffle furnace (Model: MF-1700C, Best Equipment, Shanghai, China), RH was immediately heated to temperatures of 600 °C, 700 °C, and 800 °C for the combustion tests. This involved a heating gradient of 10 °C/min sustained for 2 h, succeeded by a temperature reduction of 30 °C at a cooling rate of 20 °C/min, culminating in the acquisition of RHA. The resultant material was then subjected to a milling protocol in a planetary ball mill (Model: QXQM-10, Ten Can Powder, Shenzhen, China). Parameters set for the milling operation included a feed rate of 10 g, 300 revolutions per minute (rpm), an intermittent milling time of 10 min, and an aggregate milling duration of 4 h.

### 2.3. Characterization of RHA

For each treatment method, 0.5 g of the sample was transferred to a centrifuge tube, to which 10 mL of deionized water was added. Each tube underwent ultrasonication for 10 min to ensure homogeneity, before particle size measurement was undertaken. D50 (in nm) and polydispersity index (PDI) of the prepared silica nanoparticles were measured using dynamic light scattering (DLS) with a Malvern Zetasizer (Model: Nano-ZS90, Malvern Instruments Ltd., Moorburn, UK) maintained at 25 °C. Each sample measurement was repeated three times. Between samples, the test chamber underwent a thorough cleaning with acetone to prevent cross-contamination.

Following ball milling of RHA, nanoparticle morphology was examined using a Zeiss EVO 18 (Model: Zeiss, MERLIN Compact, Wiefelstede, Germany) scanning electron microscope (SEM) under low vacuum conditions. Given the inherently non-conductive nature of the composite samples, a fine gold layer was applied to each sample surface to ensure conductivity during SEM analysis.

X-ray diffraction (XRD) patterns were acquired using a Bruker MSAL XD2 X-ray Diffractometer (Model: Bruker, Karlsruhe, Germany), operated with CuKα radiation at settings of 40 kV and 30 mA. Scans were conducted over a 2-theta range from 10° to 80°. For data interpretation and structural pattern elucidation, EVATM Software (http://www.evasoftwaresolutions.com/) was employed.

Characterization of functional groups and chemical bonding of RHA was carried out using FTIR (Model: Nicolet, Thermo F.S., Erlangen, Germany). Potassium bromide was used as a dispersant, and the FTIR samples were prepared via the pressing method. In order to prevent the free water adsorbed on the sample surface from interfering with the spectra, the sample material should be dried in an oven for more than 10 h before sample preparation, and the whole sample preparation should be carried out under a heat preservation heating lamp. The scanning rate was 5 cm^−1^/s, and the wavelength range was 400~4000 cm^−1^.

### 2.4. Purity Analysis of Silica in RHA

The silica content in RHA was determined according to the industry standard “HG/T 3062-3072-2008” [34] of the People’s Republic of China. The specific method was as follows:

(1) The RHA sample was first dried in the blast dryer at 105 ± 5 °C for 12 h.
(2) We weighed 2 g of the dried RHA sample (noted as m_1_, accurate to 0.1 g), placed it in a customized platinum crucible, and scorched it until the weight no longer changed, recording this mass as m_2_.
(3) We prepared hydrofluoric acid 40 wt%, 98% concentrated sulfuric acid, diluted 1:1 with water.
(4) A small amount of distilled water was used to moisten the cauterized sample, then 15 mL of hydrofluoric acid was measured using a measuring cylinder and 1 mL of sulfuric acid was pipetted into a platinum crucible, and then the sample was heated and cauterized to a slurry.
(5) We cooled the crucible and collected the residue in the center of the crucible, continued to add 10 mL of hydrofluoric acid and heat evaporation to dry it, then placed it into the muffle furnace at 1000 °C for 15 min.
(6) We removed the crucible and cooled it to room temperature, and measured and recorded the mass of the crucible and residue m_3_.

The silica content (%) in RHA is calculated using the formula:

(1) $$w\left(SiO_{2}\right)=\frac{m_{2}-m_{3}}{m_{1}}\times 100$$

where m_2_ represents the total mass of the sample and the customized platinum crucible burned to constant weight in air; m_3_ denotes the total mass of the final residue and the customized platinum crucible at the end of the burn; and m_1_ represents the total mass of the sample after drying.

### 2.5. Nomenclature for Experimental Conditions

For this study, the following naming conventions were established for clarity:

(1) Pretreatment methods: acid leaching (H), boiling (Z), and water leaching (L).
(2) Drying methods: oven drying (H) and microwave drying (W).
(3) Torrefaction temperatures: 210 °C (210), 270 °C (270), and 330 °C (300).
(4) Combustion temperatures: 600 °C (600), 700 °C (700), and 800 °C (800).

Given the above conventions, a sample designation like HW210/600 would be interpreted as a sample that underwent acid washing, was dried using microwaves, torrefied at 210 °C for 40 min, and subsequently pyrolyzed at 600 °C for 2 h.

### 2.6. Parametric Optimization

For economic efficiency, energy consumption, and to save time in the experiment, the Taguchi method with an L9 (2^2^ × 3^3^) orthogonal hybrid array was employed for the experimental design. Four critical factors were considered: drying (A), combustion (B), roasting (C), and the pretreatment method (D). The control factors and their respective levels are shown in Table 3.

Table 4 illustrates the experimental conditions, as determined by the experimental design, alongside their corresponding D50 values and signal-to-noise (S/N) ratio. To facilitate calculations and minimize computational errors, the original D50 data were modified. This involved dividing the original values by 100, rounding the results, and then subtracting 6.

Subsequently, the experimental results were translated into S/N ratios, where the S/N ratio signifies the influence of changes in input variables on the output response. Minitab 19.0 software (Model: Minitab 19.0, Minitab, PA, USA) aided the design and analysis of the experiment, enabling the generation of plots showing the primary means and S/N effects [12]. For S/N ratio, the minimum D50 relationship was calculated under the “smaller is better” criterion and is defined as follows:

(2) $$\frac{S}{N}=-10\log\left(\frac{1}{n}\sum_{i=1}^{n}y_{i}^{2}\right)$$

where y_i_ denotes the response for characteristics, and *n* denotes the number of experimental scenarios.

## 3. Results

### 3.1. Data Analysis

#### 3.1.1. Visual Analysis

Table 5 shows the range analysis of the D50, as processed using Minitab 19. In analyzing the D50 response values, it is evident that the D50 of the fourth, fifth, and seventh tests falls below 800 nm among the nine test groups. Notably, the seventh group registered the lowest D50 value of 576 nm. This group’s combination was A2B1C3D2, which translates to a torrefaction temperature of 310 °C, a combustion temperature of 600 °C, boiling water demineralization, and microwave drying. The D50 values for the other test groups ranged between 800 nm and 1400 nm.

#### 3.1.2. Range Analysis

Range analysis, often abbreviated as the R method, is a common tool for interpreting the results of orthogonal experiments. A factor’s influence on the test performance index depends on its R value, which increases as the analysis progresses. The range of the D50 in the experimental results was analyzed to study the influence of various factors on its particle size. Polar difference analysis relied on the experimental D50 values for the effect of each factor on the particle size. The implication here is that a smaller D50 corresponds to a smaller average particle size and hence, in this context, a smaller D50 is more desirable.

**Figure 1:**
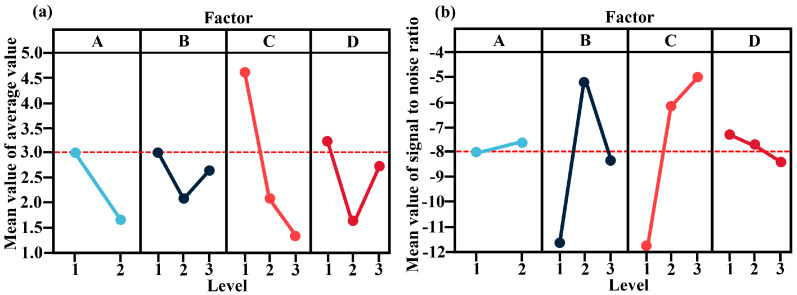
Main effects plots: (**a**) the mean of D50 and (**b**) the S/N ratio for D50.

Table 5 reveals that the extreme values for each factor, in descending order, appear in the order CDAB. Factor C’s extreme value is the largest, surpassing the extreme differences of the other three factors by 179.6%, 225.6%, and 300%, respectively. This indicates that factor C (the torrefaction temperature) has the greatest impact on the response value D50. The optimal combination, as illustrated in Figure 1a, is A2B2C3D2, consistent with the notion of minimizing the value.

Concurrently, as depicted in Figure 1b, substantial S/N differences were observed for factors B and C, while the variations for factors A and D appeared relatively consistent. Generally, a higher S/N ratio represents a factor of greater significance. The optimal combination was determined to be A2B2C3D2. This translates to torrefaction at 330 °C, combustion at 700 °C, boiling water decontamination, and microwave drying. The red dotted line in the Figure 1 only represents the middle line of the ordinate and has no other meaning. Evidently, the only variation between the optimal combinations derived from the extreme difference analysis and the direct observation was in the level of factor B. The extreme difference analysis was B2 (700 °C), while the direct observation indicated B1 (600 °C). Variations in the initial furnace temperature and the surface humidity of both the tank and the ball during the ball milling test might have contributed to the above results when the sample was introduced to the muffle furnace.

#### 3.1.3. Variance Analysis

Analysis of variance (ANOVA) stands as a prevalent statistical tool for processing experimental results [35]. This tool was used to determine the factors that had a significant impact on the D50 and to assess whether the discrepancies in test outcomes across each factor level were attributable to test errors or the variations within the factor levels themselves [36]. The D50 results from the RH combustion median size test were analyzed using Minitab.19, and the outcomes are displayed in Table 6.

Based on F and *p* values (Table 6), it becomes evident that, while none of the factors were significant, factor C (the torrefaction temperature) exhibited the greatest effect on the D50. The remaining factors, in decreasing order of influence, are: A (the drying method), D (the pretreatment method), and B (the combustion temperature), with factor B exerting the least influence. Except for factor C, which was significantly more impactful, the significance levels of the other three factors on the D50 exhibited marginal variances. As a result, when comparing the relevance ranking of the relevance of the factors (from large to small), the sequence CADB (derived from ANOVA) aligns closely with CDAB (obtained from the extreme variance analysis).

Synthesizing insights from the three analysis perspectives, the consensus on the descending order of each factor’s significance on D50 is: CDAB. The optimal combination was A2B2C3D2, translating to torrefaction at 330 °C, combustion at 700 °C, boiling water decontamination, and microwave drying.

### 3.2. Effects of Different Treatment Methods on RHA Size and Dispersion

**Figure 2:**
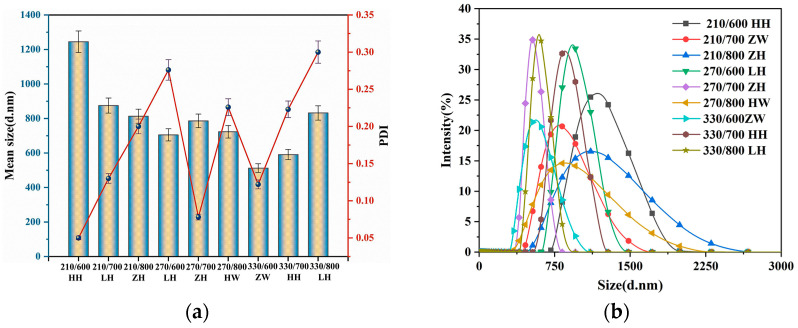
(**a**) Average size and PDI of RHA for RH after different treatment methods. (**b**) Typical particle size distribution pattern of RHA.

Figure 2a illustrates the average size and PDI of RH after different RHA treatment methods. According to the previous analysis, factor C (torrefaction temperature) exerted the most substantial influence on the D50. In contrast, the effects of the other three factors were marginal and comparable. In Figure 2a, which zeroes in on pertinent parameters, the smallest D50 aligns with a torrefaction temperature of 330 °C. In contrast, the broadest D50 dispersion corresponds to a torrefaction temperature of 210 °C. When averaging three test sets for each torrefaction temperature, the mean values associated with 210 °C, 270 °C, and 330 °C were 1041 nm, 813.7 nm, and 710.3 nm, respectively. As the torrefaction temperature escalates, the D50 consistently diminishes, although this reduction in particle size is also gradually declining. This is attributed to the pronounced decrease in oxygen content in torrefied biomass, resulting in an effective increase in calorific value and grindability. Such enhancements favor ensuing gasification and combustion processes. The torrefaction temperature reached 330 °C, surpassing the conventional temperature range utilized for biomass torrefaction (200–300 °C). Such deviations can be ascribed to modifications in the hull sample’s composition post roasting, such as hemicellulose decomposition and the partial depolymerization of cellulose and lignin [37]. Additionally, the RH sample’s internal structure underwent significant alterations due to the cross-linking carbonization reactions of the carbonized RH [20]. Consequently, RH’s cellulose underwent cross-linking, carbonization, and partially decomposition, and there was a marked surge in inorganic mineral components, predominantly SiO_2_. In summary, these modifications, induced via the heightened torrefaction temperatures during RH combustion, are pivotal in refining the subsequent grinding particle size of RHA.

PDI is a dimensionless number deduced from the autocorrelation function. It provides insight into the particle size distribution based on parameters obtained from DLS. Generally, a PDI value ranging from 0.1 to 0.5 is indicative of reliable measurements and well-formed colloidal suspensions. A PDI nearing 0.7 suggests that the sample might be inconsistently distributed in the solution, potentially containing large particles or aggregates with a relatively broad size distribution. Figure 2a reveals that, for the RHA samples in this study subjected to varying treatment methods, the PDI values spanned from 0.05 to 0.31. Accordingly, with water serving as the medium, and following the ultrasonic treatment method, the RHA dispersion was relatively uniform. As depicted, the prevalence of large particles or aggregations was virtually negligible, leading to a tighter particle size distribution. Consequently, these metrics offer a more authentic representation of RHA’s particle size distributions.

Figure 2b illustrates the typical particle size distribution patterns of RHA obtained from RH subjected to various treatment methods. It is evident from the curves that there are differences in peak height and width values depending on the treatment methods. The 210/600 HH group exhibited the most prominent peak coupled with the narrowest peak width. In contrast, the 330/800 LH group manifested the widest peak width. These observations imply that the particle size distribution for the 210/600 HH group is tightly clustered with minimal dispersion. In contrast, the 330/800 LH group showed a more dispersed distribution, aligning with the extreme PDI values in Figure 2a.

Figure 2b shows typical RHA particle size distribution patterns acquired in RH us-ing various treatment procedures. The curve illustrates that different treatment procedures have distinct peak heights and peak widths. The peak of the 210/600 HH group was the most important, with the smallest peak width. The peak width was greatest in the 330/800 LH group. These findings indicate that the particle size distribution of group 210/600 HH is densely packed and has a low dispersion. The distribution of the 330/800 LH group, on the other hand, is more spread out, which is consistent with the PDI extremes in Figure 2a.

### 3.3. Verification of Significant Factor

**Figure 3:**
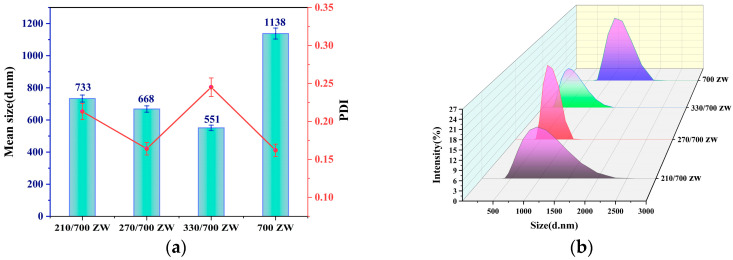
(**a**) Average size and PDI of RHA obtained at different torrefaction temperatures. (**b**) Typical particle size distribution pattern of RHA.

According to the Taguchi method and ANOVA, among the four different factors, only factor C (torrefaction) has a significant effect on the D50 response, and the other three factors have marginal differences in significance regarding the D50 response. Therefore, verification tests were performed to prove the significance of factor C. The optimal combination obtained via the Taguchi method is A2B2C3D2, which is calcined at 330 °C, with combustion at 700 °C, decontamination by boiling water, and drying using microwaves. A set of single-factor validation tests was designed according to the optimal combination. After the rice husk samples were boiled and microwave-dried, the combustion temperature at 700 °C was kept unchanged, and only the combustion temperature was changed. The tests were labeled as 210/700 ZW, 270/700 ZW, and 330/700 ZW. In addition, a control group was set up, the samples were calcined directly at 700 °C after boiling and microwave drying, and the RHA was labeled as 700 ZW. The resulting RHA was then subjected to ball milling. Finally, the particle size distribution of RHA-SiO_2_ was measured, and the test results are shown in Figure 3.

Figure 3a shows that the average size of RHA particles before torrefaction is 1138 nm, but the average size of particles after torrefaction varies from 551 nm to 733 nm. The average particle size of the control group 700 ZW was 2.06 times larger than that of 330/700 ZW. In conclusion, torrefaction can significantly enhance the abrasiveness of RH-based silica and noticeably reduce the average particle size. Furthermore, we observed a gradual decrease in the average size of RHA-SiO_2_ particles with an increasing torrefaction temperature, accompanied by an expanding range of reduction. These findings indicate that increasing the torrefaction temperature enhances the grindability of Rh-based silica, facilitating the production of silica with a smaller particle size.

The PDI values of SiO_2_ particles obtained after torrefaction at various temperatures range between 0.15 and 0.25. This indicates that the dispersion of RHA-SiO_2_ particles is more favorable at a combustion temperature of 700 °C. The control group exhibited the lowest PDI value (700 ZW), while the high-temperature torrefaction group with 330/700 ZW showed the highest PDI value, suggesting that the high-temperature torrefaction treatment had minimal impact on the dispersion of RHA-SiO_2_ particles.

Figure 3b depicts the typical particle size distribution of RHA produced via the torrefaction of RH at various temperatures. The waveform graph clearly shows that the waveforms produced after torrefaction at different temperatures, 210 °C, 270 °C, and 330 °C, exhibit significant changes in wave crest and wave breadth. The 270/700 ZW category has the highest visible wave crest and the smallest wave width. The 210/700 ZW group has the widest wave width. The 700 ZW group’s wave crest is more visible, and the wave breadth is rather small. These findings suggest that torrefaction at a medium temperature can lower the size distribution range of RHA-SiO_2_. Torrefaction at low and high temperatures has minimal influence on the particle size distribution.

### 3.4. Effect of Different Treatment Methods on the Purity of RHA-SiO_2_

**Figure 4:**
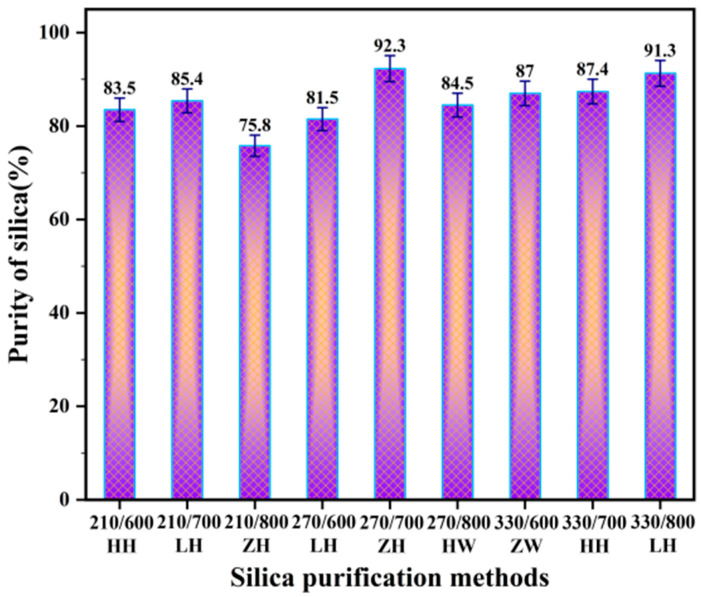
The purity of RHA obtained via different treatment methods.

Figure 4 shows the silica content of RHA obtained via different treatment methods. Obviously, the combination with the highest silica content is 210/700 ZH, followed by 330/800 LH; the corresponding silica concentrations are 92.3% and 91.3%. The remaining combinations have silica concentrations below 88%. The combination with the lowest silica concentration is 210/800 ZH, with only 75.8%. It may be because, with the low-temperature torrefaction of RH at 210 °C, only hemicellulose is decomposed by heat, and the decomposition level of cellulose and lignin is relatively small, or there is no decomposition. Both hemicellulose and cellulose are thermally decomposed under medium temperature torrefaction at 270 °C. Due to the relatively mild decomposition environment, the cross-linking carbonization of cellulose occurs less. When the temperature rose rapidly to 800 °C after the end of torrefaction, the cross-linking carbonization of cellulose occurred, and the decomposition reaction promoted the formation of inorganic minerals. The acidic groups (carboxyl and phenol groups) contained in lignin decompose and deprotonation occurs under this condition, meaning that the lignin is negatively charged and adsorbent to metal ions. The above reaction reduces the purity of the silica.

Figure 4 also reveals that when the C (torrefaction temperature) component alone is taken into account, the average purity of SiO_2_ corresponding to different torrefaction temperatures of 210 °C, 270 °C, and 330 °C is 81.6%, 86.1%, and 88.6%, respectively. This means that the purity of SiO_2_ tends to increase as the torrefaction temperature rises, but the rate of increase decreases dramatically after the temperature approaches 270 °C. Similarly, when only the D (pretreatment temperature) component is considered, the average purity of SiO_2_ at 600 °C, 700 °C, and 800 °C is 84.0%, 88.4%, and 83.9%, respectively. It shows that the purity of SiO_2_ in RHA increases first and then decreases with the increase in calcination temperature. And the purity of silica reaches the maximum at the calcination temperature of 700 °C.

### 3.5. Effect of Different Treatment Methods on RHA’s Crystal Structure

**Figure 5:**
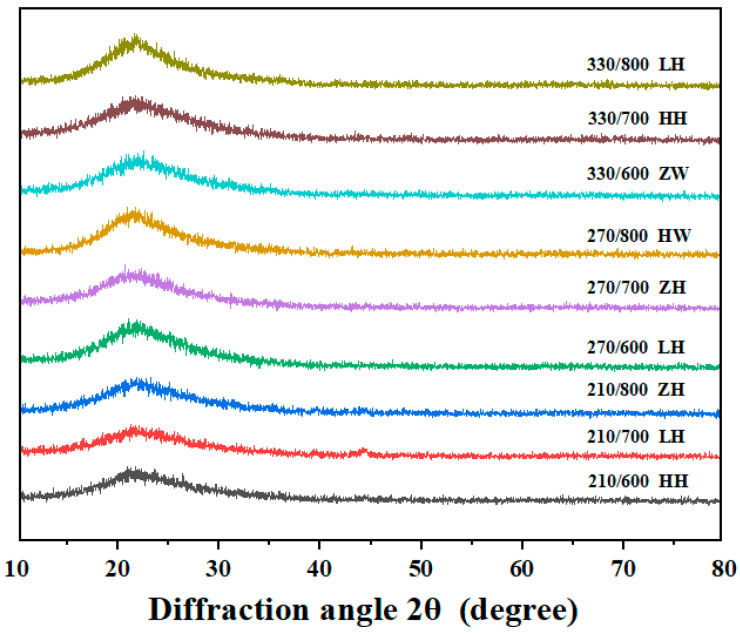
X-ray of RHA obtained via different processing methods.

Figure 5 presents the XRD of RHA derived from various treatment methods of RH. Several observations can be drawn from the displayed patterns. First, across all nine sample groups, a broad diffusion peak with a maximum intensity near 2θ ≈ 22.5° dominates the profiles [17]. The absence of other distinct crystallographic peaks and the general similarity across patterns suggest that the primary crystal structure of RHA remains significantly unchanged across different treatment methods [38]. Second, these patterns closely mirror the standard spectrum of amorphous SiO_2_, confirming the primary constituent of the resultant product as amorphous SiO_2_ [39,40]. Third, the diffraction intensity for samples treated at a combustion temperature of 800 °C was more pronounced than its counterparts at lower temperatures. This suggests that at temperature below 750 °C, Si-based powder produced from RH combustion remains predominantly amorphous. Furthermore, the pretreatment method seemed not to impact the crystalline state of the product. A critical observation is the sensitivity of the amorphous-to-crystalline phase transition of RHA-based SiO_2_ to combustion temperatures exceeding 700 °C. The intensity of the characteristic peak at 800 °C underscores that the transition from amorphous to crystalline phases in RHA SiO_2_ is initiated once the combustion temperature crosses 750 °C.

### 3.6. Effect of Different Treatment Methods on RHA Functional Groups

**Figure 6:**
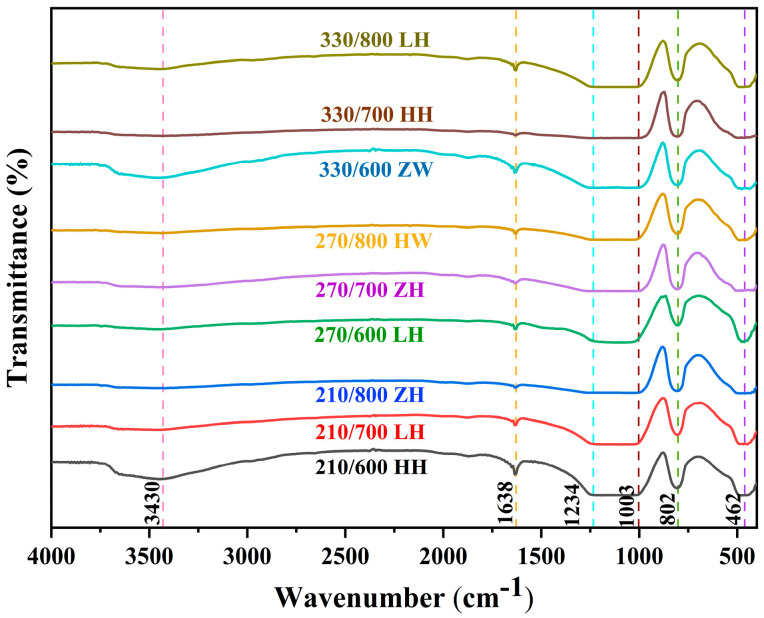
FTIR spectra of RHA obtained via different processing methods.

The combined Fourier-transform infrared spectrum (FTIR) is shown in Figure 6. The broadband around 2500–3600 cm^−1^ was attributed to O–H stretching in RH-based silica, which confirms the presence of hydroxyl group at the silica nanoparticles’ surface and the water absorption in samples [41,42]. The FTIR spectra of RH-based silica samples treated in different ways showed continuous absorption peaks at 1003, 802, and 462 cm^−1^ [39,43]. In brief, the vibration signal at 1003 cm^−1^ was the asymmetric stretching of Si–O–Si. The peaks at 802 cm^-1^ and 462 cm^−1^ represented the symmetric stretching and asymmetric bending of the Si-O-Si bond, respectively [43,44]. These Si–O–Si stretching and bending phenomena were confirmed silica characteristics. Moreover, the peak at 1638 cm^−1^ was the H-O-H bending vibration peak of water.

### 3.7. Effect of Different Treatment Methods on RHA Morphology

**Figure 7:**
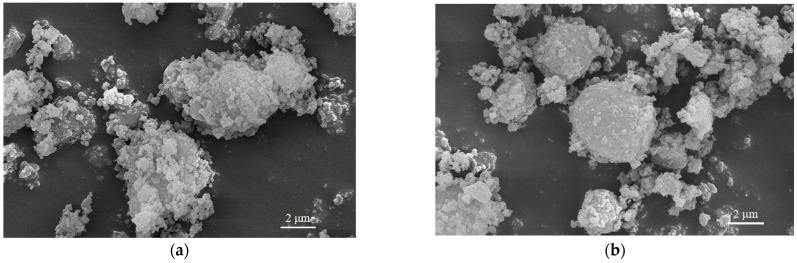

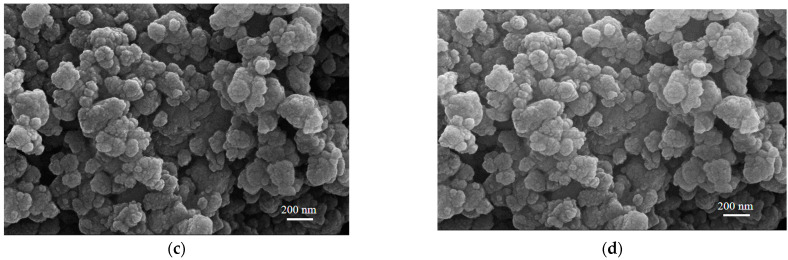
Scanning electron microscope images of 210/600 HH (**a,c**) and 330/600 ZW (**b,d**) at different magnifications.

Figure 7 depicts the morphological variations in RHA samples from two different treatment methods: 210/600 HH and 330/600 ZW. The choice of these treatment methods, with a significant difference in their D50 values, facilitates a clearer comparison of particle sizes. The magnified morphology of RHA particles (Figure 7c,d) shows the agglomerates of SiO_2_ nanoparticles [45]. Moreover, the SiO_2_ agglomerates in the 330/600 ZW treatment method (Figure 7b) manifest a less dense configuration and feature relatively smaller particle sizes. Such observations might be attributed to the RH undergoing an early transition to the slow combustion phase at a low temperature due to the elevated roasting temperature used. Additionally, microwave drying, with its demonstrated efficiency of being over 30 times faster than traditional drying methods, aids in biomass surface disruption and enhances volatility, while reducing crystallinity [46]. This quickened drying method, in turn, results in a decrease in the oxygen concentration of RH, accelerates cellulose carbonization, and induces partial lignin degradation. Collectively, these modifications contribute to the improved grindability of RHA.

## 4. Conclusions

Utilizing the Taguchi approach, it was discovered that torrefaction was the optimal pretreatment method preceding the conversion of RH via combustion to RHA, exerting a significant effect on the SiO_2_ particle size distribution. The optimal combination, A2B2C3D2, corresponded to a torrefaction temperature at 330 °C, combustion at 700 °C, a boiling step, and microwave drying, and this combination facilitated the attainment of the minimum D50 value. When combustion was executed at temperatures not exceeding 800 °C, XRD analysis of RHA consistently yielded an amorphous SiO_2_ powder. The FTIR spectrum of prepared RH-based silicon represented the absorption band that confirmed silica characteristics.

Validation tests confirmed that torrefaction treatment can enhance the abrasiveness of SiO_2_ derived from RH. Furthermore, increasing the torrefaction temperature reduced the average particle size substantially. According to the results of silica concentration tests carried out on the RHA samples, the purity of the silica positively correlates with the torrefaction temperature. RHA with a silica content of up to 92.3% was created. Consequently, this study elucidates the significant factors governing the synthesis of nanometer SiO_2_, positioning RH as an eco-friendly SiO_2_ source.

## Figures and Tables

**Table 1:** Proximate and ultimate analysis of RH samples.

Name	Proximate Analysis (wt%)			Ultimate Analysis (wt%)				
	V	A	FC	C	H	N	S	O
RH	59.6	24.8	15.6	43.5	6.4	0.6	0.1	35.2

Note: In the table, V represents the volatile content of the sample. A represents the ash content of the sample. FC represents the fixed carbon of the sample. C, H, N, S, and O represent the carbon, hydrogen, nitrogen, sulfur, and oxygen in the sample, respectively.

**Table 2:** RH pretreatment method processes.

Processing Method	Raw Material	Instrument/Reagent	Process
Acid leaching	RH	H2SO_4_ 0.5 mol/L	RH at room temperature with a solid–liquid ratio of 1:20, soak for 2.5 h, and then rinse with water to neutral, drain, bag for standby
Boiling	RH	Electric cooker/water	Boil 1 L of water in an electric pot, add 50 g of RH, boil continuously for 2.5 h (keep adding water during this time), and then rinse with water, drain, bag, and set aside
Water leaching	RH	Water	Rinse RH with running water at room temperature, drain, and bag
Microwave	Pretreated RH	PM2003 microwave oven	Take 50 g of the treated RH each time, put it in a microwave oven, heat it for 7 min, and adjust the power to 450 W
Drying	Pretreated RH	Blast dryers	Take out 50 g of treated RH each time, put it into a blast-drying oven at 105 ± 5 °C, and dry continuously for 24 h until a constant weight is reached
Torrefaction	RH after secondary drying	Muffle furnace/crucible	A 15 g sample was placed in a crucible and, when the muffle temperature was raised to 50 °C, the crucible was placed, and then the temperature was raised to 210 °C, 270 °C, and 330 °C for 40 min of torrefaction, with a temperature rise rate of 10 °C/min

**Table 3:** Control parameters with levels.

Control Factor	Level			
	Ⅰ	Ⅱ	Ⅲ	Units
A: Drying method	Oven drying (H)	Microwave drying (W)	—	—
B: Combustion treatment	600	700	800	°C
C: Torrefaction treatment	210	270	330	°C
D: Pretreatment method	Acid leaching (H)	Boiling (Z)	Water leaching (L)	—

**Table 4:** Design of the experiment and the results.

Exp. No.	Drying Method A (—)	Combustion Treatment B (°C)	Torrefaction Process C (°C)	Pretreatment Method D (—)	Original Data D50 (nm)	Treated Data D50 (nm)	PDI
1	1 (H)	1 (600)	1 (210)	1 (H)	1316	7	0.098
2	1 (H)	2 (700)	2 (270)	2 (Z)	849	2	0.135
3	1 (H)	3 (800)	3 (330)	3 (L)	902	3	0.331
4	1 (H)	1 (600)	2 (270)	3 (L)	782	2	0.310
5	1 (H)	2 (700)	3 (330)	1 (H)	653	1	0.247
6	1 (H)	3 (800)	1 (210)	2 (Z)	876	3	0.231
7	2 (W)	1 (600)	3 (330)	2 (Z)	576	0	0.161
8	2 (W)	2 (700)	1 (210)	3 (L)	931	3	0.164
9	2 (W)	3 (800)	2 (270)	1 (H)	810	2	0.260

**Table 5:** D50 range analysis table of mixed orthogonal test results.

Exp. No.	Drying Method A (—)	Combustion Treatment B (°C)	Torrefaction Process C (°C)	Pretreatment Method D (—)	Treated Data D50 (nm)
1	1 (H)	1 (600)	1 (210)	1 (H)	7
2	1 (H)	2 (700)	2 (270)	2 (Z)	2
3	1 (H)	3 (800)	3 (330)	3 (L)	3
4	1 (H)	1 (600)	2 (270)	3 (L)	2
5	1 (H)	2 (700)	3 (330)	1 (H)	1
6	1 (H)	3 (800)	1 (210)	2 (Z)	3
7	2 (W)	1 (600)	3 (330)	2 (Z)	0
8	2 (W)	2 (700)	1 (210)	3 (L)	3
9	2 (W)	3 (800)	2 (270)	1 (H)	2
K1	18	9	13	10	
K2	5	6	9	5	
K3	/	8	4	8	
k1	3.00	3.00	4.33	3.33	
k2	1.67	2.00	3.00	1.67	
k3	/	2.67	1.33	2.67	
R	1.33	1.00	3.00	1.67	
Primary and secondary factors	C > D > A > B				
Optimal combination	A2	B2	C3	D2	

**Table 6:** The variance calculation results of the D50 mixed orthogonal test.

Variance Source	DF	Seq SS	Adj SS	Adj MS	F	*p*
Drying method A	1	3.556	3.556	3.556	0.59	0.582
Combustion treatment B	2	1.556	1.556	0.778	0.13	0.891
Torrefaction process C	2	14.889	14.889	7.448	1.24	0.536
Pretreatment method D	2	4.222	4.222	2.118	0.35	0.766
Error	1	6.000	6.000	6.000		
Amount	8	30.222				

Note: Seq SS represents the continuous sum of squares as a measure of the variance from the different sources listed in the model. Adj SS represents the adjusted sum of squares as a measure of the variance from the different sources listed in the model. Adj MS is the adjusted mean square which measures the degree to which a term or model explains variability. The total degree of freedom (DF) represents the amount of information in the data. The F-value is the test statistic used to determine whether any term in the model is associated with the response. The *p*-value is a probability that measures the evidence against the null hypothesis.

## Data Availability

Data are contained within the article.
